# Facile Synthesis and High Photocatalytic Degradation Performance of ZnO-SnO_2_ Hollow Spheres

**DOI:** 10.1186/s11671-016-1740-y

**Published:** 2016-11-28

**Authors:** Changqing Jin, Chenghai Ge, Zengyun Jian, Yongxing Wei

**Affiliations:** School of Materials and Chemical Engineering, Xi’an Technological University, Xi’an, 710021 People’s Republic of China

**Keywords:** ZnO-SnO_2_, Hollow spheres, Templates, Photocatalytic degradation, Heterostructure, Mesoporous

## Abstract

ZnO-SnO_2_ hollow spheres were successfully synthesized through a hydrothermal method-combined carbon sphere template. The samples were characterized by X-ray diffraction (XRD), scanning electron microscopy (SEM), transmission electron microscopy (TEM), and Fourier transform infrared spectroscopy (FT-IR). The average diameter of hollow spheres is about 150 nm. The photocatalytic activity of the as-prepared samples was investigated by photodegrading Rhodamine B. The results indicated that the photocatalytic activities of ZnO-SnO_2_ hollow spheres are higher than ZnO hollow spheres. The degradation efficiency of the hollow spheres could reach 99.85% within 40 min, while the ZnO hollow spheres need 50 min.

## Background

With the rapid development of society, water pollution has been becoming a growing environmental problem in the world. The photocatalytic degradation of organic pollutants in water using semiconductors, such as TiO_2_, ZnO, and SnO_2_, has attracted increasing attention in the past two decades [1–7]. Among the various semiconductor oxides, ZnO has been considered as one of great potential materials because of their high photocatalytic activity, no-toxicity, chemical stability, and relatively low cost [8–10].

However, the high recombination rate of the photogenerated electron-hole pairs limits the photocatalytic efficiency. Currently, coupled semiconductor photocatalysts are regarded as the effective method to enhance the photocatalytic activity by increasing the charge separation and extending the photo-responding range [11–16]. As we know, there are many researches on the coupled ZnO-SnO_2_ photocatalysts [17–20]. Lin et al. synthesized coupled ZnO/SnO_2_ photocatalysts by co-precipitation method, which reveals high photocatalytic activity for the degradation of methylene blue [17]. Chen et al. successfully prepared ZnO/SnO_2_ hetero-nanofibers via an electrospun method using zinc chloride and stannous chloride as inorganic sources, and the ZnO/SnO_2_ hetero-nanofibers exhibit an enhanced photodegradation ability to congo red [18]. But there are few reports about synthesizing ZnO-SnO_2_ hollow spheres by using carbon spheres as templates.

In this study, we report a simple environmental-friendly method for the preparation of ZnO-SnO_2_ hollow structures by using carbon spheres as templates. In addition, the photocatalytic property of ZnO-SnO_2_ hollow spheres is investigated.

## Methods

### Synthesizing Carbon Spheres

The carbon spheres were prepared by hydrothermal method. First, 6.44-g glucose was dissolved in 65-ml deionized water under magnetic stirring for 20 min. Then, the solution was transferred to a 100-ml Teflon-lined stainless steel autoclave and the autoclave was reacted at 180 °C for 10 h. After hydrothermal reaction, the precipitate was washed by centrifuging with ethyl alcohol and deionized water and dried in air at 80 °C for 6 h.

### Preparing ZnO-SnO_2_ Hollow Spheres, ZnO Hollow Spheres

ZnO-SnO_2_ hollow spheres were synthesized by carbon sphere template. 0.20-g carbon spheres and 0.5 g Zn(CH_3_COO)_2_·2H_2_O and 0.5 g SnCl_4_·5H_2_O were dissolved in 40-ml ethyl alcohol by ultrasonication for 30 min. Then, the suspension was magnetically stirred for 12 h at room temperature, followed by centrifuging repeatedly with ethanol. Finally, the obtained products were dried at 80 °C for 5 h and calcined in air at 500 °C for 4 h with the heating rate of 4 °C/min.

The ZnO hollow spheres were synthesized by the same method with the addition of 1.0 g Zn(CH_3_COO)_2_·2H_2_O.

### Characterization of Samples

The crystal structure of the samples were determined by an X-ray diffraction (XRD, XRD-6000), operated at 40 kV and 30 mA using Cu Kα radiation (*λ* = 0.15406 nm) at a scanning rate of 4° min^−1^ ranging from 15° to 85°. The functional groups of carbon spheres were studied by Fourier transformed infrared spectrum (FT-IR, Nexus). The morphologies and size of the products were analyzed by scanning electron microscopy (SEM, Quanta 400F) and transmission electron microscopy (TEM, JEM-2010).

### Photocatalytic Activity Measurements

The photocatalytic activity was evaluated by degradation of Rhodamine B (RhB). In a typical process, 20-mg sample was added to 20-ml RhB (10 mg/L) aqueous solution. The suspension was magnetically stirred in the dark for 30 min in order to reach adsorption/desorption equilibrium. Then, the suspension containing RhB and photocatalyst was irradiated under a high-pressure mercury lamp (CHF-XM-300W) with continuous stirring. During the experiment, analytical samples were taken out from the reaction suspension after at 10-min intervals and centrifuged to remove the particles. The RhB concentration in the supernatant was analyzed by a spectrophotometer (KD-723).

## Results and Discussion

**Fig. 1:**
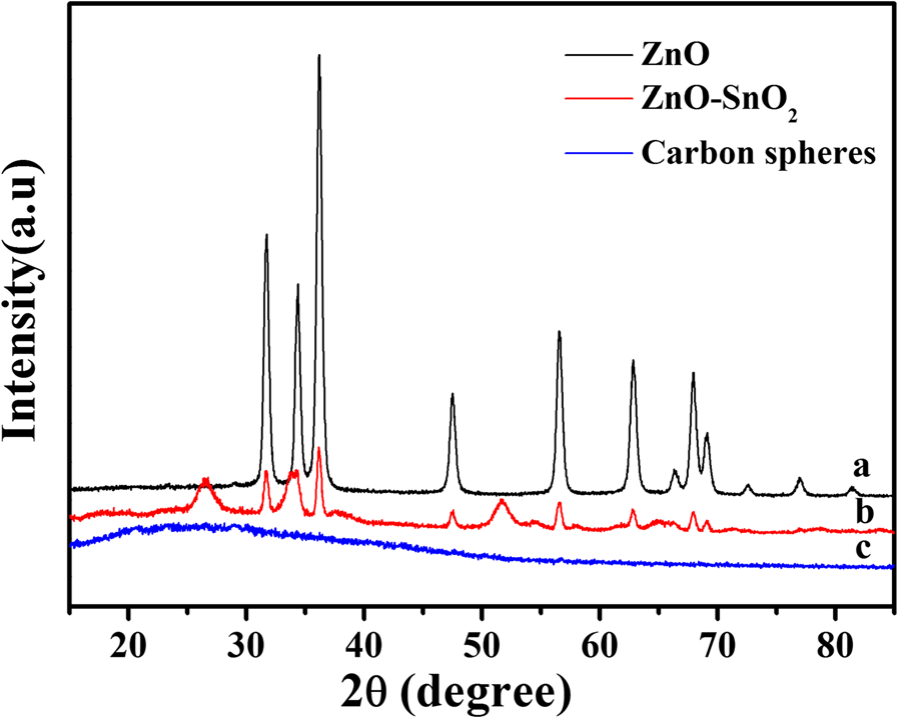
XRD patterns of samples

The crystal structures are identified by means of XRD analysis. Figure 1 shows XRD patterns of the as-prepared samples. It can be seen that the dispersing diffraction peak at about 25° of the 2*θ* appeared for the carbon spheres, which could be related to the formation of the amorphous carbon, as shown in Fig. 1c. After calcination at 550 °C for 3 h, carbon sphere was oxidized into gas to flow out. The pattern of the ZnO-SnO_2_ hollow spheres (Fig. 1b) exhibits two sets of diffraction peaks. One set of diffraction peaks is indexed to the hexagonal wurtzite ZnO phases (space group - P63/mc, JCPDS card no. 36-1451). The other set of peaks is connected to the tetragonal rutile SnO_2_ phases (space group - P42/mnm, JCPDS card no. 41-1445). No evidence of impurities is detected, indicating the as-prepared sample has a heterostructure; compared to the pattern of ZnO hollow spheres (Fig. 1a), its diffraction peaks are obviously broadened, indicating that the crystal size becomes smaller.

**Fig. 2:**
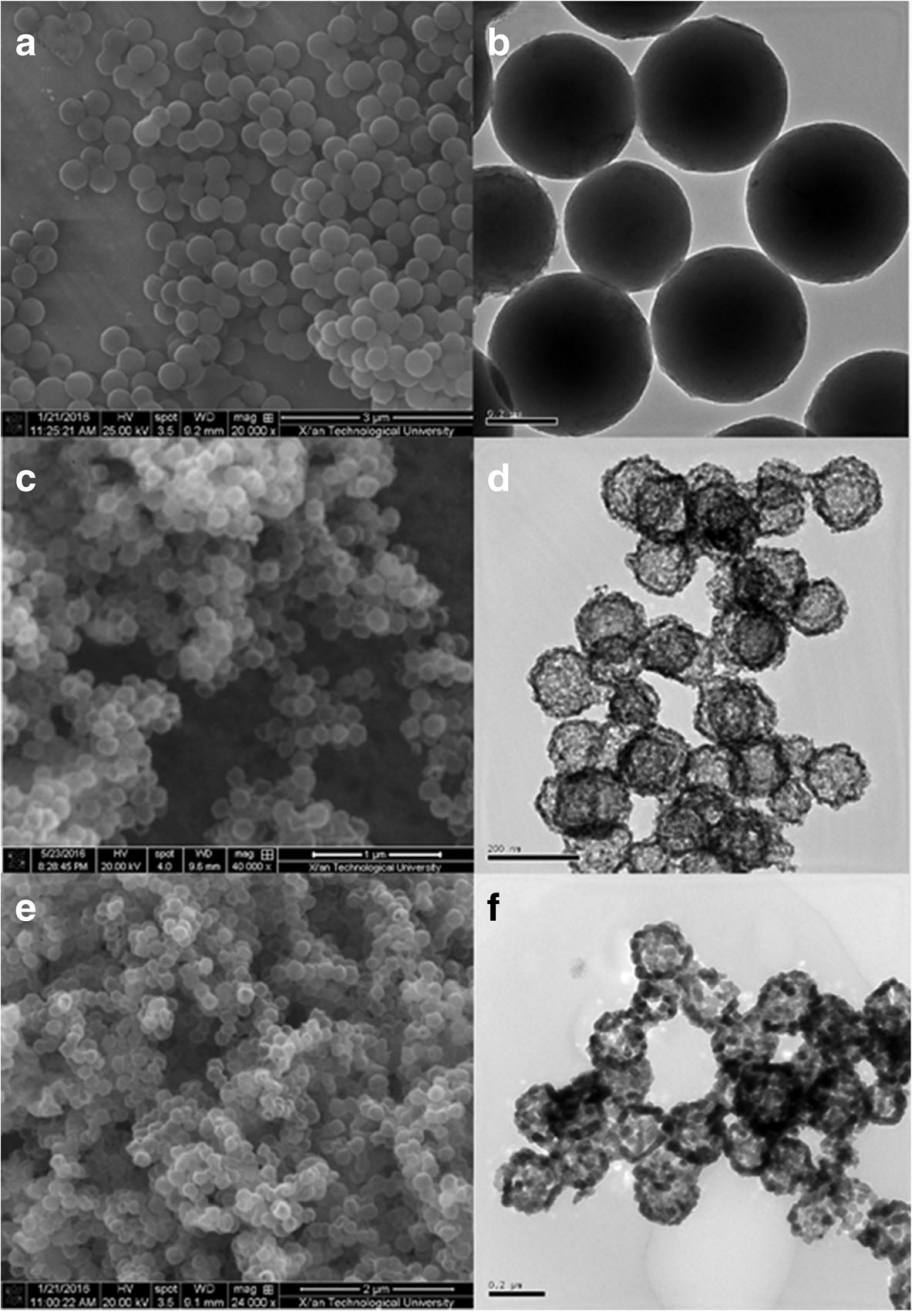
SEM images of carbon spheres (**a**), ZnO-SnO_2_ hollow spheres (**c**), and ZnO hollow spheres (**e**). TEM image of carbon spheres (**b**), ZnO-SnO_2_ hollow spheres (**d**), and ZnO hollow spheres (**f**)

In order to get more information about the templates and hollow structures, the obtained samples are further investigated by SEM and TEM, as shown in Fig. 2. Figure 2a, b shows SEM and TEM images of carbon spheres prepared via a typical hydrothermal treatment. Apparently, all of the carbon spheres exhibit very similar spherical morphology with uniform morphology. It can be seen that the average diameters of carbon spheres are about 400 nm. Figure 2c, d shows numerous ZnO-SnO_2_ hollow spheres with a diameter of about 150 nm. It is obviously found that the diameter is smaller than its templates. The shrinkage could be attributed to the decarbonization and dehydration of carbon spheres during the process of calcination, and carbon sphere is oxidized into gas to flow out. Compared to the ZnO hollow spheres (Fig. 2e, f), ZnO-SnO_2_ hollow spheres have a smaller crystal size about 10 nm, indicating that ZnO-SnO_2_ heterostructure prepared through adding extra SnCl_4_·5H_2_O could inhibit the grain growth. It is easy to understand that the nucleation and crystal growth of ZnO (or SnO_2_) facilitates heterogeneous nucleation of SnO_2_ (or ZnO). Subsequently, crystal growth of SnO_2_ (or ZnO) suppresses growth of ZnO (or SnO_2_), which is caused by growth competition. As is suggested by TEM and XRD results, the ZnO-SnO_2_ heterostructures have smaller particle and crystal size than ZnO hollow spheres.

**Fig. 3:**
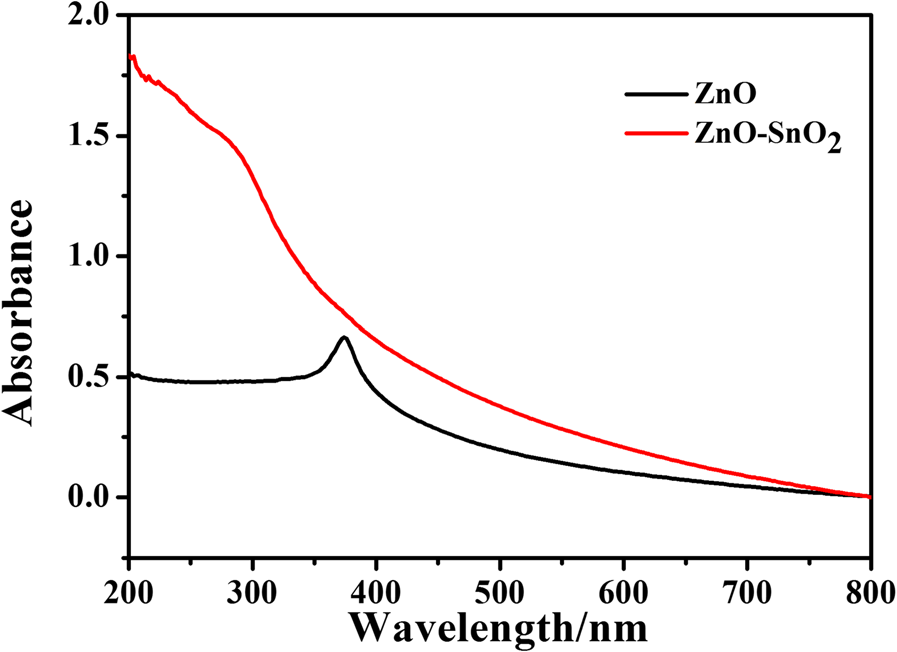
The UV–vis spectra of ZnO and ZnO-SnO_2_ hollow spheres

The UV–vis absorption spectra of ZnO and ZnO-SnO_2_ hollow spheres are shown in Fig. 3. It can be seen that the wavelength of the absorption edge for ZnO hollow spheres is located at 375 nm, which can be attributed to the intrinsic absorption band derived from the band gap transition. The obtained ZnO-SnO_2_ hollow spheres showed much stronger absorbance intensities and larger absorbance region than ZnO hollow spheres. This result indicates that the formation of heterostructure is beneficial for photocatalytic performance in the UV and visible light regions [21].

**Fig. 4:**
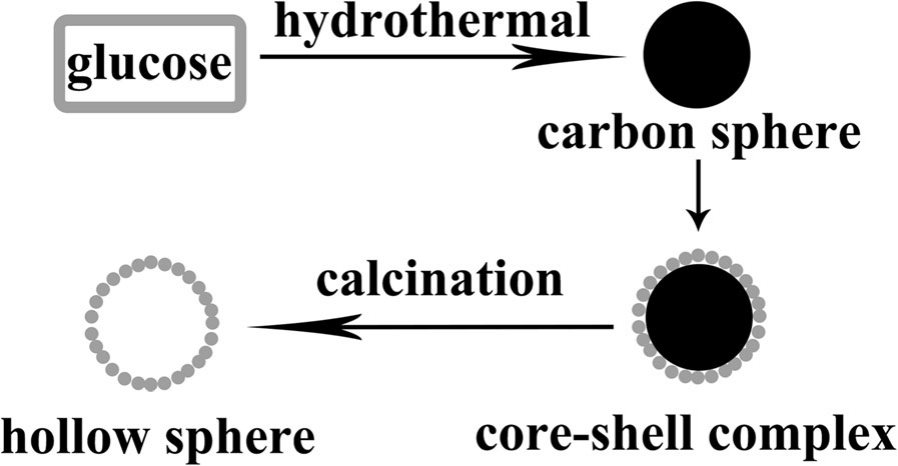
Schematic illustration of the formation of hollow spheres

The formation of ZnO and ZnO-SnO_2_ hollow spheres involves using carbon spheres as template, and the schematic illustration of the formation is demonstrated in Fig. 4.

**Fig. 5:**
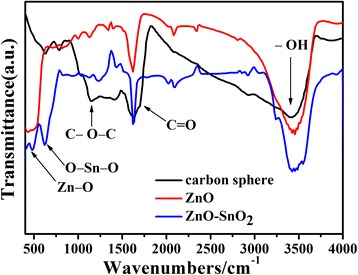
FT-IR spectra of samples

During the hydrothermal process, the growth of carbon spheres follows the LaMer model [22]. As shown in Fig. 5, the as-prepared carbon spheres have a large number of functional groups in the surface such as –OH, C=O, which is conducive to the adsorption of metal ions to form core-shell composite spheres [23, 24]. Then calcined in air at 550 °C, the hollow spheres can be obtained by the oxidation of carbon spheres into CO_2_ simultaneously and precursor with metal ions translates into metallic oxide. With further analysis of FT-IR spectra, it can be seen that the ZnO bond is assigned to the stretching frequency at 435 cm^−1^ for pure ZnO which is shifted to higher frequency as 476 cm^−1^ for ZnO-SnO_2_ hollow spheres. In addition, the weak band at 633 cm^−1^ for ZnO-SnO_2_ hollow spheres is assigned to O–Sn–O bond, which is consisted with XRD results.

**Fig. 6:**
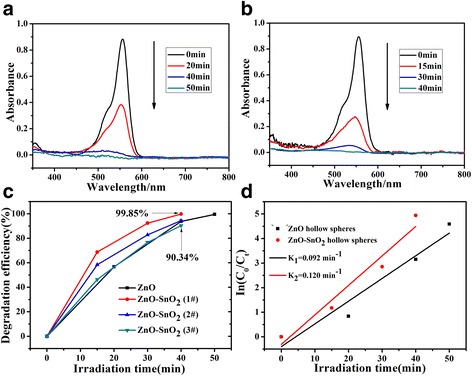
UV–vis spectra changes of RhB on the ZnO hollow spheres (**a**) and ZnO-SnO_2_ hollow spheres (**b**). Photocatalytic degradation efficiencies of RhB with ZnO hollow spheres and ZnO-SnO_2_ hollow spheres (**c**). Kinetics curves of RhB (**d**)

Photocatalytic activity of the ZnO hollow spheres and ZnO-SnO_2_ hollow spheres was examined by using RhB degradation. The degradation efficiency can be expressed as follows:

1 $$ \eta =\frac{C_0-{C}_t}{C_0}\kern0.5em \times \kern0.5em 100\%=\frac{A_0-{A}_t}{A_0}\kern0.5em \times \kern0.5em 100\% $$

where *C*_0_ is the initial concentration of RhB and *C*_*t*_ represents the concentration after *t* min reaction. UV–vis spectra of RhB in contact with different catalyst samples after 30-min dark adsorption/desorption are shown in Fig. 6a, b. Figure 6c shows that the degradation efficiency of the RhB (20 mg/L) in aqueous catalyst dispersion with ZnO-SnO_2_ hollow spheres could reached 99.85% within 40 min while the ZnO hollow spheres need more time. The high degradation efficiency of the RhB could be related to the heterostructure and the reduced crystal size.

In order to further investigate the photocatalytic activities of these catalysts, pseudo-first-order kinetics are used to analyze the photocatalytic degradation kinetics:

2 $$ \ln \frac{C_0}{C_t}=kt $$

where *k* is the kinetic rate constant (min^−1^) and *t* is the irradiation time (min). The relationship curves of ln(*C*_0_/*C*_*t*_) versus irradiation time are plotted in Fig. 6d. The kinetic rate constant of ZnO hollow spheres and ZnO-SnO_2_ hollow spheres are 0.092 and 0.120 min^−1^, respectively. Meanwhile, the ZnO-SnO_2_ hollow spheres used in the photocatalytic activity measurements are centrifuged, dried at 80 °C for 3 h before it is reused as such in the succeeding photocatalytic experiment. It can be seen that ZnO-SnO_2_ hollow spheres exhibit excellent reusability in photocatalytic degradation of RhB, and the degradation efficiency could reach 90.34% after used two times (Fig. 6c). These results indicate that the ZnO-SnO_2_ hollow spheres have higher photocatalytic activities than ZnO hollow spheres. Due to the presence of the ZnO-SnO_2_ heterostructure, the probability of the recombination of electron-hole pairs is significantly reduced and the photo-responding range is extended [12–15]. In addition, the unique hollow nanostructure with mesoporous spheres provides efficient molecular transport pathways to their interior surface, increases the catalyst surface area, and provides more reaction site for photodegrading RhB.

## Conclusions

In this paper, mesoporous ZnO-SnO_2_ hollow spheres were successfully synthesized by using a hydrothermal method-combined environmental-friendly carbon sphere template. ZnO-SnO_2_ hollow spheres with a diameter of about 150 nm have smaller particle and crystal size than ZnO hollow spheres. The degradation efficiency of the RhB with ZnO-SnO_2_ hollow spheres could reach 99.85% within 40 min while the ZnO hollow spheres need more time in the same indications. The results indicate that the ZnO-SnO_2_ hollow spheres are good photocatalysts. It could be inferred that the heterostructure can effectively inhibit the recombination rate of the photogenerated electron-hole pairs and extend the photo-responding range. Furthermore, they can be well applied in sensors, photo-electric materials, and so on.

## Abbreviations

FT-IR: Fourier transform infrared spectroscopy
RHB: Rhodamine B
SEM: Scanning electron microscopy
TEM: Transmission electron microscopy
XRD: X-ray diffraction
